# Application of a Sequential Extraction Method for Analyzing Cu Distribution in Pre-Treated Mine Tailings after Electrodialytic Remediation

**DOI:** 10.3390/ijerph16040584

**Published:** 2019-02-18

**Authors:** Andrea Lazo, Henrik K. Hansen, Pamela Lazo, Claudia Gutiérrez

**Affiliations:** 1 Departamento de Ingeniería Química y Ambiental, Universidad Técnica Federico Santa María, Avenida España 1680, Valparaíso 2390123, Chile; henrik.hansen@usm.cl (H.K.H.); claudia.gutierrez@usm.cl (C.G.); 2 Instituto de Química y Bioquímica, Facultad de Ciencias, Universidad de Valparaíso, Avenida Gran Bretaña 1111, Playa Ancha, Valparaíso 2360102, Chile; pamela.lazo@uv.cl

**Keywords:** copper, electrodialytic remediation, mine tailings, sequential extraction

## Abstract

Mine tailings have been analyzed by a sequential extraction procedure after their pre-treatment with a leaching solution for 24 h and electrodialytic remediation during 15 days with a constant electric field of 2.7 V cm^−1^. Four leaching solutions were tested: H_2_SO_4_ + HNO_3_ (2:1 vol.) pH = 1.9; H_2_SO_4_ + HNO_3_ (2:1 vol) pH = 4.2; NH_4_Cl 0.8M, pH = 5.5 and 30% H_2_O_2_ adjusted to pH 2 with HNO_3_ 1M + HCl 1M. After the treatment, the tailings were divided in six slices from anode to cathode. The highest removal efficiency of copper was obtained with H_2_SO_4_ + HNO_3_ pH = 1.9, which allows one to remove 67% of the copper in the total cell and 85% of the copper in the slice closest to anode. The same solution with pH = 4.2 allows one to remove 62% of the total copper. The analysis realized by the sequential extraction method indicates the easy removal of water-soluble and exchangeable fractions in all experiments, moreover, residual and sulfide are the less mobile fractions. The general trend was the movement of copper associated to different fractions from anode to cathode and its accumulation closest to the cathode in the case of exchangeable, Fe-Mn oxides and acid soluble fractions, possibly due to some precipitation of copper compounds associated with less acidic conditions.

## 1. Introduction

Mine tailings have become a focus of attention due to their environmental impacts. In Chile, tailings are mainly associated to the copper sulfide concentration process and to a lesser extent to the concentration of gold minerals. They are a mixture of fine-grained ground-up rock and process water with dissolved metals and reagents that remain after the minerals of economic importance have been extracted [1].

Electrokinetic remediation is a technique which allows the removal of heavy metals from soil, sediments and sludge, by application of a low intensity direct current (DC) electric field. The electric field induces the movement of heavy metals through the porous matrix towards the electrodes. Some advantages of electrokinetic remediation include the possibility of using this technique in soils with low permeability, in situ remediation and low cost compared to other traditional remediation technologies, however, its practical application has limitaions, such as lower removal efficiencies and longer treatment times [2]. Among these techniques, electrodialytic soil remediation includes the use of ion exchange membranes to separate soil and processing solutions. Several chemical reactions are induced by the application of electric fields, where the descomposition of water at the electrodes is the most important one, producing oxygen gas and hydrogen ions. The pH of the soil and its changes affects the movement of contaminants being decisive in the removal of heavy metals [3,4]. In the last 20 years, both methods have proven to be ways to remove heavy metals from fine-grained porous solids and there are a series of studies about their application to mine tailings [5,6,7].

Chemical elements occur in Nature in different forms and their toxicity depends on this. Over the past four decades, chemical sequential extraction has been an interesting method to partition the total metal content and determine how heavy metals are associated with different soil components [8,9,10]. Previous studies using contaminated soils indicated the importance of pH and the type of soil in the results of sequential extraction analysis after electrodialytic process [11]. Analysis by sequential extraction of four fractions was carried out after the pre-treatment of tailings with a particular solution and electrodialytic remediation, but no conclusive results were obtained about the distribution of fractions through the cell [12].

In the present work, tailings were pre-treated with different chemical solutions to leach copper ions. In mining industry, the most used solution to leach copper is sulfuric acid. Nitric acid is also an energetic oxidant whose behavior depends on its concentration and temperature. Occasionally, the leaching of sulfide minerals requires an oxidant like ferric ion, ferric chloride or ferric sulfide in acidic media [13,14,15,16]. For oxidized copper ores, ammonium chloride has also been used [17,18]. The aim of this pre-treatment is to achieve decrease the pH in the case of acid solutions or complex formation in the case of ammonium chloride solution; in both cases the removal of copper would favor its dissolution [12].

Electrodialytic remediation experiments were carried out after the pre-treatment of the tailings with different leaching solutions. A six fractions chemical sequential extraction method was used to analyze and determine the associations of copper in the mining tailing, throughout the electrochemical cell, with the aim to understand the effect of particular pre-treatment solutions and the application of an electric field in the removal of certain fractions associated with copper.

## 2. Materials and Methods

### 2.1. Electrodialytic Remediation

Four electrodialytic remediation experiments were carried out with a mine tailing obtained from El Teniente copper mine located in O`Higgins Region of Chile. Before the electrodialytic treatment the mine tailing was pre-treated for 24 hours with one of the following solutions: solution 1, H_2_SO_4_ + HNO_3_ (2:1 vol.) pH = 1.9; solution 2, H_2_SO_4_ + HNO_3_ (2:1 vol) pH = 4.2; solution 3, NH_4_Cl 0.8M, pH = 5.5 and solution 4, 30% H_2_O_2_ adjusted to pH 2 with HNO_3_ 1M + HCl 1M. Each solution was mixed with the tailing to obtain a humidity near to 20%.

The used reagects were sulfuric acid 95–97% (ISO grade, Merck, Kenilworth, NJ, USA); ntric acid 65% (Ph Eur, ISO, Merck); ammonium chloride (ACS, Ph Eur, ISO, Merck, Kenilworth, NJ, USA); 30% hydrogen peroxide (ISO, Merck, Kenilworth, NJ, USA); hydrochloric acid 37% (ACS, Ph Eur, ISO, Merck, Kenilworth, NJ, USA). All reagents were analytical grade.

Electrodialytic experiments were carried out at room temperature with a constant electric field of 2.7 V cm^−1^ and the current was recorded on-line. The duration of each experiment was 21,600 min (15 days).

A custom-made acrylic cell [12] of 0.05 m diameter with a central compartment of 212 mL volume was used. The length of the central compartment was 0.08 m and the total length (with anolyte and catholyte compartment) was 0.15 m. The volume of the anolyte and catholyte compartments was 144 mL. An anion and a cation exchange membrane from Membranes International Inc. (Ringwood, NJ, USA) corresponding to CMI-7000 and AMI-7001 were used. The anode and cathode were made of titanium.

H_2_SO_4_ 0.5 N and H_2_SO_4_ 0.005 N were used as catholyte and anolyte solutions, respectively. The solutions were recirculated using Model MD-6-230GSO peristaltic pumps (Iwaki, MA, USA). The data were measured online with a model UT60A digital multimeter (UNI-T, China).

After each experiment, the tailings were divided into six equal sized slices throughout the cell, each one with a thickness of 0.013 m. All slices were analyzed by the sequential extraction method [19,20]. Total copper was measured in the slices closest to the anode and closest to the cathode, humidity and pH were measured in each slice for all experiments. All measurements were carried out on three replicate samples and their average values, with a standard deviation less than 5%, are presented here.

### 2.2. Sequenial Extraction Method

This procedure separates heavy metals into six fractions: water-soluble, exchangeable, acid soluble bound, Fe-Mn oxides bound, organic and sulfide bound and residue [19,20]. In the case of tailings, due to the origin, no organic matter or carbonate are expected in the composition, and for this reason the fractions will be called sulfide fraction and acid soluble fraction, the latter corresponding to the fraction extractable at pH 5.0 [20,21]. The details of the procedure are presented below:

Soluble fraction: 1 g of dry sample was extracted by centrifugation for 2 h with 15 mL of deionized water at room temperature.

Exchangeable fraction: The residue of soluble fraction was extracted with 8 mL of 1M MgCl_2_ at pH 7 for 1 h at room temperature.

Carbonate-bound fraction and/or extractable at pH = 5.0: The residue of exchangeable fraction was extracted with 8 mL of NaOAc (adjusted to pH = 5.0 with HOAc) by stirring for 5 h at room temperature.

Iron and manganese oxide-bound fraction: The residue of the previous fraction was extracted with 15 mL of 0.04 M NH_2_OH·HCl in 25% *v*/*v* HOAc at 96 °C with occasional stirring during 6 h at room temperature.

Organic matter and sulfide fraction: The residue of previous fraction was extracted with 3 mL of 0.02 M HNO_3_ and 5 mL of 30% H_2_O_2_ (adjusted to pH = 2.0 with HNO_3_). The mixture was heated to 85 °C during 3 h with occasional stirring. A 3 mL aliquot of 30% H_2_O_2_ (pH = 2.0 with HNO_3_) was added and the mixture was heated to 85°C during 3 h with occasional stirring. When the sample was cool, 5 mL of NH_4_OAc in 20% *v*/*v* HNO_3_ were added, the samples were diluted to a final volume of 20 mL and agitated continuously for 30 min.

Residual fraction: The residue of previous fraction was digested with a solution of HCl-HNO_3_, with stirring during 16 hours at room temperature.

The reagents used were magnesium chloride hexahydrate (Ph Eur, ISO, Merck); hydroxylamine hydrochloride (ACS, 98%, Merck, Kenilworth, NJ, USA); anhydrous sodium acetate (ACS, Ph Eur, Merck, Kenilworth, NJ, USA). The other reagents were listed above.

### 2.3. Total Copper Measurement

The total content of copper in the tailings was measured according to Danish Standard Ds. 259 (1982) “Determination of metals in water, sludge and sediments—general guidelines for determination by atomic absorption spectrometry in flame” using a SpectrAA spectrometer according to EPA Method 9045 (Varian, CA, USA).

### 2.4. pH Measurement

pH was measured according to EPA Method 9045.

### 2.5. Moisture of Tailings

Moisture was determined by weight loss until constant mass (24 h) after drying in a oven at 105 °C.

## 3. Results and Discussion

The copper content in the original tailing was 1109 of Cu/kg dry tailing, humidity of 0.7% and pH of 3.9. The sequential extraction analysis was carried out for the initial sample; the concentrations of copper associated to each fraction in mg of Cu/kg of dry tailing are presented in Table 1. The composition of tailings shown 40% of total copper associated to not adsorbed copper or easily soluble copper compounds (soluble fraction), 31% of secondary and primary sulfides (sulfide fraction), 17% associated to copper silicates or crystallized compounds (residual fraction), and lower percentages associated to the other fractions.

**Figure 1:**
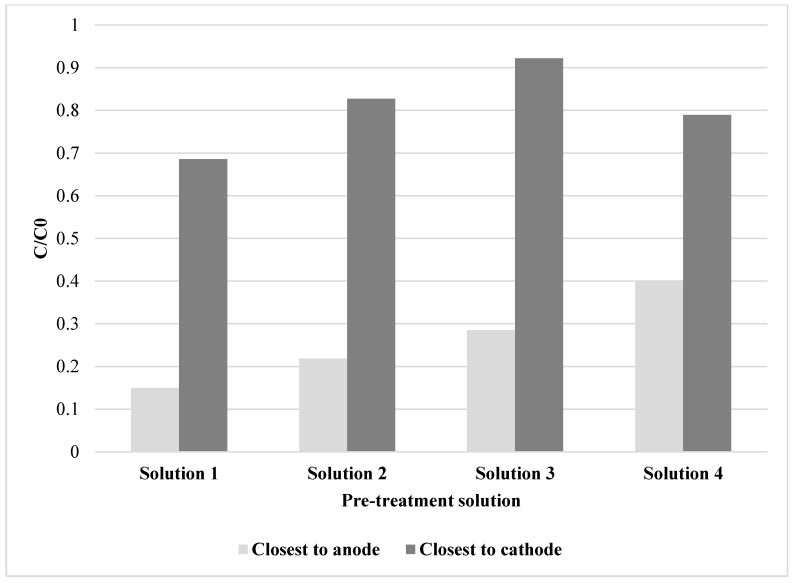
C/C_0_ ratio of copper concentration for slices closest to anode and closest to cathode for different pre-treatment solutions after electrodialytic remediation.

Figure 1 shows C/C_0_, where C corresponds to the concentration of copper at the end of experiment for a particular slice and C_0_ is the value of copper concentration at the beginning of the experiment for the same slice, both values are shown as a C/C_0_ ratio and, in this case, concentrations are presented for the slices closest to cathode and closest to the anode, for each pre-treatement solution after electrodialytic remediation.

In all experiments the slice closest to the anode showed a great decrease in copper concentration after the application of the leaching solution and electrodialytic treatment. The highest removal efficiency was obtained with H_2_SO_4_ + HNO_3_ 2:1 *v*/*v* pH = 1.9 pre-treatment solution, with a reduction of copper concentration higher than 80%. The copper concentration in the slice closest to the cathode was relatively close to the initial concentration in all experiments, due to the movement of copper ions through the cell from anode to cathode and its accumulation close to the cathode, clearly, the mobility of copper ions was strongly influenced by pH.

Respect to the total copper removal in the slice closest to the anode, efficiencies of 85% and 78% were obtained in the case of H_2_SO_4_ + HNO_3_ at pH 1.9 and 4.2, respectively. A 71% removal was achieved when the tailing was pre-treated with NH_4_Cl 0.8M and finally, the pre-treatment with hydrogen peroxide solution allowed the removal of 60% of the total copper in this fraction. The removal efficiencies calculated for the slices closest to the cathode were certainly no more than 31%, mainly due to the accumulation of copper mentioned above.

**Figure 2:**
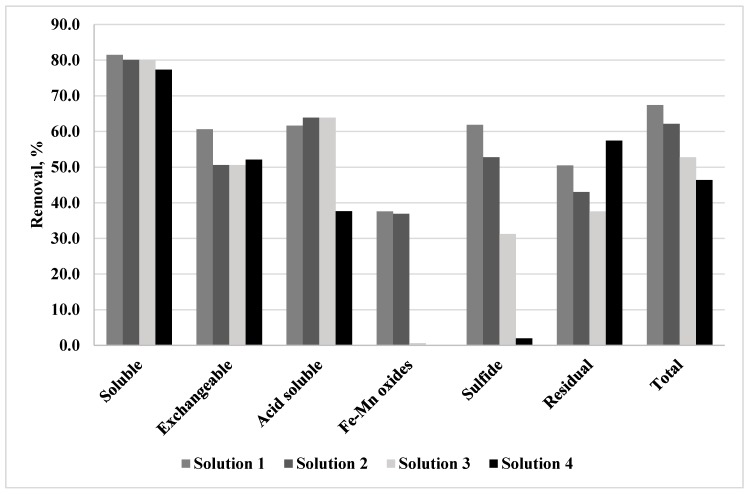
Global copper removal efficiencies for each fraction and pre-treatment solution from anode to cathode.

The copper removal efficiencies calculated for each fraction and each pre-treatment solution after electrodialytic experiments are shown in Figure 2, where global removal efficiencies are also presented. From Figure 2 it is possible to observe that the global removal efficiencies, for the complete cell, are 67.4% and 62.1% with pre-treatment solutions of H_2_SO_4_ + HNO_3_ with pH = 1.9 and pH = 4.2, respectively; 46.4% with the solution containing H_2_O_2_, while with NH_4_OH 0.8M the total Cu removal was 52.8%.

**Figure 3:**
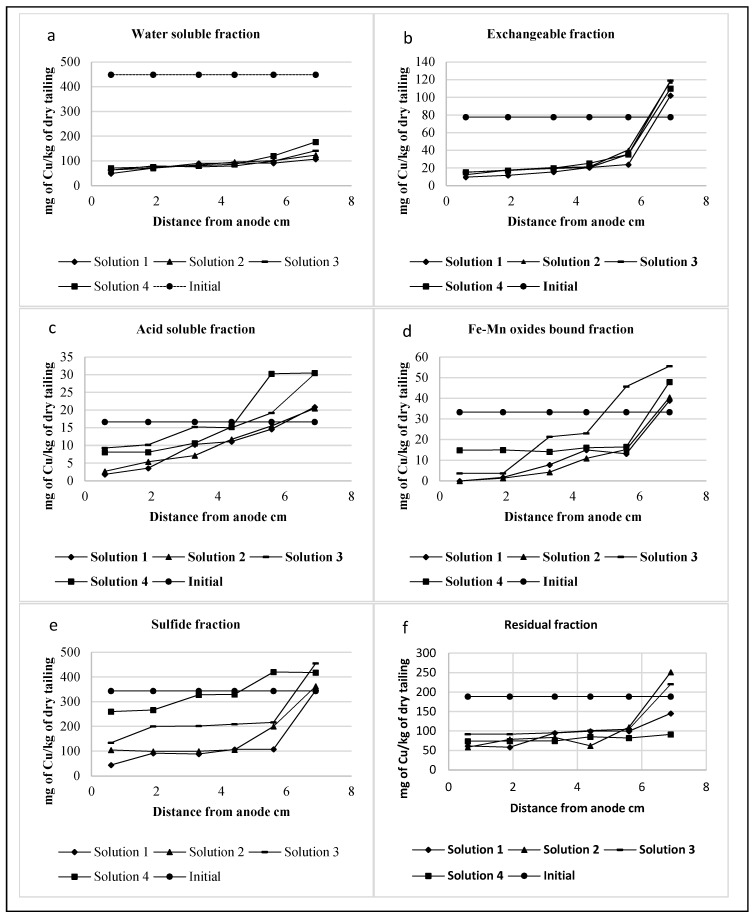
Copper associated to (**a**) Water-soluble fraction; (**b**) Exchangeable fraction; (**c**) Acid soluble fraction; (**d**) Fe-Me oxides bond fraction; (**e**) Sulfide fraction; (**f**) Residual fraction.

**Figure 4:**
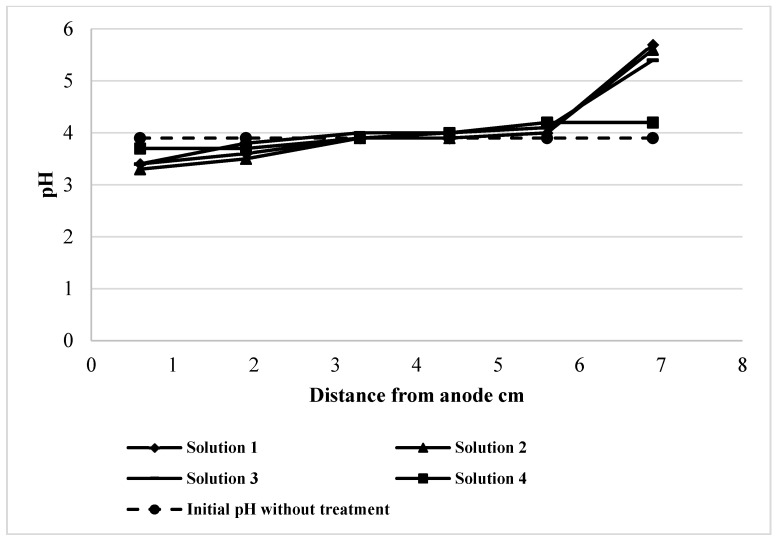
pH evolution from anode to cathode after electrodialytic remediation.

The copper concentrations associated with each fraction from anode to cathode are presented in Figure 3. The general trend indicates the highest removals in the case of the water soluble, exchangeable and Fe-Mn bound fractions and lowest removals of copper associated to sulfide and residual fractions in the slice closest to the anode. Certainly, the mobility of copper ions was linked to the pH trend through the cell, which is shown in Figure 4. A quick removal associated to low pH is observed near the anode, where the mobility and the dissolution of copper were enhanced, while on the other hand, the accumulation of metal or its reduced mobility with the pH increase due to the generation of hydroxyl ions, produced lower removal efficiencies.

These results corroborate those reported by Ottosen et al., [22] who observed an accumulation of the released copper towards the cathode in the section close to the anode and associated this accumulation zone with the evolution of pH, thus indicating the importance of this parameter in the process.

For pre-treatment solutions with sulfuric and nitric acid, the copper ions associated to Fe-Mn oxides bound practically disappear from the slices closest to the anode. Exchangeable, water-soluble and acid soluble fractions show a great decrease, with the remaining copper concentration associated to these fractions being less than 20% of the initial concentration in almost all cases. The sequential extraction procedures assume the decrease in mobility and bioavailability from water-soluble to residual fraction, i.e., residual fraction is the least mobile. In the present study, the analysis indicated that the sulfide and residual fractions are the less mobile fractions and, in this case. the pH value has an important role allowing a much greater release of copper associated with the residual fraction in the case of the solution with hydrogen peroxide and pH 2 [21].

Specifically, in the case of the water-soluble fraction, the reduction in copper concentration was between 61% to 89% in the complete cell depending on the pre-treatment solution, as can be seen in Figure 3a. The biggest reductions were obtained for pre-treatment solution of H_2_SO_4_ + HNO_3_. This fraction has a high environmental impact, because it is the more mobile fraction and could easily be released.

The exchangeable fraction shows a significant reduction in all slices except in the one closest to the cathode, where an accumulation of copper associated to this fraction is observed, indicating the movement of cations from the anode to the cathode. Figure 3b shows clearly the movement of copper associated with the exchangeable fraction and its accumulation near the cathode.

The Fe-Mn oxides fraction and the fraction of copper extractable at pH = 5.0 show the same behavior as the exchangeable fraction, with a clear depletion of copper associated to these fractions towards the anode. In both cases, there is a great accumulation of copper in the slice closest to the cathode, as shown in Figure 3c,d, possibly due to the precipitation of Cu-Fe-Mn oxides and hydroxides or salts because of the pH increase [23,24].

The concentration of copper mainly associated with the exchangeable, Fe-Mn oxides and acid soluble fractions increased from the anode to the cathode and, in almost all cases, an accumulation in the slice closest to the anode occurred. The accumulation of copper ions in the slice closest to the cathode in these cases highlighted the movement of copper ions associated with the fractions and can be correlated to the development of an acidic front in the soil from the anodic membrane to the cathode produced by water dissociation [11,22].

As Figure 3e illustrates, in the case of the sulfide fraction, higher removals were obtained with pre-treatment solutions containing sulfuric acid and nitric acid, as their lower pH allows a better performance, in these cases the mobility of copper associated to this fraction from anode to cathode is evident. The removal of this fraction with the pre-treatment solutions of NH_4_Cl and H_2_O_2_ was very low compared to H_2_SO_4_ + HNO_3_, obtaining 39% and 76% of removal in the slice closest to the anode, respectively.

The residual fraction, as Figure 3f shows, undergoes a higher removal towards the anode, which can be associated to the decrease in pH, which possibly alters the distribution of copper [24].

In all experiments, the evolution of pH through the cell shows clearly its increase towards the cathode, which a decrease in the mobility of copper ions and probably promoting the precipitation of certain compounds. In the slice closest to the anode the pH is lower than the initial pH of the tailings and the pH of the slice closest to the cathode is considerably higher than the initial pH, except in the case of hydrogen peroxide solution.

## 4. Conclusions

Taking into account the complete cell, the highest removal efficiency was obtained with the pre-treatment solution of H_2_SO_4_ + HNO_3_ with pH = 1.9; this solution makes it possible to remove 85% of copper in the slice closest to anode and 67% of the total copper content in the total tailing mass.

A little lower copper removal efficiencies were obtained with the same solution with a pH = 4.2, in this case, 78% of the total copper was removed in the slice closest to the anode and 62% in the complete cell.

Among the cases studied, the lowest copper removal efficiency was obtained with the pre-treatment with hydrogen peroxide solution, with which 60% of the total copper was removed in the slice closest to thr anode and a decrease in the total concentration of copper in the complete cell close to 46% was achieved.

The analysis by sequential extraction indicates that the relative order of the fractions in the slice closest to the anode remained more or less unchanged. Moreover, residual and sulfide are the less mobile fractions. A little more of 50% of total copper was associated to more mobile fractions (water-soluble, exchangeable and acid soluble). The mobility of different fractions and their accumulation probably were linked to the pH evolution through the cell.

## Figures and Tables

**Table 1:** Initial concentration of copper in each fraction.

	Water-Soluble	Exchangeable	Acid Soluble	Fe-Mn Oxides Bound	Sulfide	Residual
Cu, mg of Cu/kg of dry tailing	449.1	77.6	33.3	16.6	343.8	188.5
